# Neonatal Resuscitation

**Published:** 2014-07-10

**Authors:** Sushmita Bhatnagar

**Affiliations:** Department of Pediatric Surgery, B.J.Wadia Hospital for Children, Mumbai

 (This section is meant for residents to check their understanding regarding a particular topic)

## Questions

1. In which circumstances is resuscitation required in a neonate?
2. What are the clinical parameters of initiating resuscitation in the newborn?
3. What are the steps and sequence in resuscitation?
4. What is “initial assessment”?
5. What factors are critical for successful resuscitation?
6. What are the parameters to be looked at while reassessing the baby receiving resuscitation?
7. What is the highest priority in neonatal resuscitation?
8. What is the technique of chest compressions while resuscitating a neonate?
9. What is the indication, route of administration and dose of epinephrine?
10. What is Refractory shock?

## Answers

**Answer 1**

AT BIRTH

- Preterm with respiratory compromise.
- Congenital anomalies with respiratory compromise – pulmonary hypoplasia, congenital diaphrag-matic hernia, etc.
- Meconium aspiration.
- Congenital cystic lung lesions.

NEONATAL PERIOD

- Shock – septic, hypovolemic, etc.
- Post-operative state.
- Deterioration of the primary pathology.

**Answer 2 **

Those newborns do not require resuscitation can generally be identified by a rapid assessment of the follow-ing 3 characteristics:

Term gestational age at birth.
Crying or breathing.
Good muscle tone.

Presence of these 3 parameters indicates that the baby is well and does not require resuscitation. Ob-servation of breathing, activity, and color should be ongoing.

**Answer 3 **

Within the first 15 minutes, the ABC must be appropriately restored and maintained after initial assessment is done and the need for resuscitation is established. (1)

A – Airway - Clear airway, suctioning of mouth, oral cavity, positioning of the neck in sniffing air po-sition, bag and mask ventilation or endotracheal intubation as required.

B – Breathing – Adequate ventilation, oxygenation and lung perfusion.

C – Circulation – Assessment of perfusion, blood pressure, heart rate followed by chest compressions in the ratio of 1:3 (one ventilation followed by 3 chest compressions). Isotonic fluids or colloids are to be given as boluses at 10cc/kg upto 60cc/kg until perfusion improves or hepatomegaly develops.

Following the ABC, other parameters, which significantly contribute to the success or failure of re-suscitation in the first 15 minutes are to be corrected. These are:

- Hypoglycemia
- Hypocalcemia
- Acidosis
- Hypothermia
- Control of Sepsis (Antibiotics)
- Patent ductus arteriosus, which causes left to right shunting (prostaglandins may be needed).

**Answer 4 **

The first one-minute (“the Golden Minute”) is the most crucial period wherein the baby is assessed for the need of resuscitation and reevaluated before ABC is initiated. The decision to progress beyond the initial steps is determined by simultaneous assessment of 2 vital characteristics:

- Respiratory rate and pattern of respiration – tachycardia, respiratory distress, labored breath-ing, apnoea, or gasping respiration, cyanosis.
- Heart rate - less than 100 beats per minute as recorded by auscultating the precordium.

**Answer 5 **

The factors which are critical for successful resuscitation are:

- Anticipation that baby might require resuscitation
- Emergency trolley preparation - as per the needs of the baby to be resuscitated with all emer-gency drugs and endotracheal tubes of 2.5, 3.0, 3.5 and 4 with the introducer available on the trolley as well as all the connectors for the ventilation mechanisms.
- Accurate evaluation – a good initial assessment allows the resuscitation process to begin be-fore the baby is in end-stage cardio-respiratory failure
- Rapid and prompt initiation of resuscitative measures by skilled personnel who can manage to ventilate the baby and give adequate and appropriate chest compressions either single handedly or with support.

**Answer 6 **

The parameters to be looked at are:

- Oxygen saturation - as measured by continuous monitoring by pulse oximeter. The disad-vantage of this non-invasive technique is that the peripheral perfusion must be good, the limbs must be warm and cardiac output must be appropriate for accurate reading.
- Capillary refill – a refill time of > 2 seconds is indicative of poor perfusion and need for further resuscitation
- Heart rate: A rise in heart rate is the most sensitive indicator of a successful response to the resuscitative techniques. An increase in heart rate should be evident by 5-10 breaths.

**Answer 7 **

Establishing effective ventilation is the highest priority and if it takes longer than 30 seconds to establish ventilation, corrective measures need to be employed. The chest compressions should not be started without first ensuring effective ventilation. 

**Answer 8 **

There are 2 techniques for effective chest compressions as regards placement of fingers, but 2 hands wrapped around chest with 2-thumb technique is preferred method. The pressure of the finger tips/thumb must concentrate over the heart and not over entire chest just above the xiphisternum. One third of the diameter of the chest must be compressed in the ratio of 1:3, i.e. 30 ventilations to 90 compressions per minute (120 events per minute).

**Answer 9 **

Epinephrine is indicated when heart rate remains less than 60/min after 30 seconds of effective ventilations and at least another 45-60 sec. of coordinated compressions and ventilations. (2,3)

The routes of administration of epinephrine available are as follows:

- Endotracheal tube – if baby is intubated, though is less effective than IV route
- Intravenous/Umbilical vein catheter – preferred method

The dose of epinephrine is 0.5-1ml/kg by ETT or 0.1-0.3ml/kg in the concentration of 1:10,000 (0.1mg/ml), which is to be followed by 0.5-1ml flush of normal saline. The heart rate should be re-checked after 1 minute of giving compressions and ventilations. The dose of epinephrine can be re-peated after 3-5 minutes if the initial dose is ineffective or can be repeated immediately if initial dose is given by endo-tracheal tube in the absence of an intravenous access.

**Answer 10 **

When the baby does not respond to the first 15-minute resuscitative measures, it is known as refractory shock. In such situations, the following protocol is to be utilized stepwise:

- Infusion of Dopamine 5-9 micrograms/kg/min and/or Dobutamine upto 10mcg/kg/min are to be initiated.
- If these drugs do not help in reversing the state of shock, epinephrine is to be added in the dose of 0.05 – 0.3 mcg/kg/min.
- In case of catecholamine resistant shock,
  - Vasodilators should be added when the blood pressure is normal and left ventricular function is poor.
  - Intravenous Milrinone/ intravenous adenosine/inhaled nitric oxide/inhaled iloprost as per the indication is to be added if blood pressure remains low and there is evidence of right ven-tricular dysfunction.
  - Norepineprine/ vasopressin/ terlipressin/angiotensin is to be added if peripheral per-fusion is good but the baby has low blood pressure.

## Footnotes

**Source of Support:** Nil

**Conflict of Interest:** The author is editor of the journal. The manuscript is independently handled by other editors and she is not involved in decision making about the manuscript.

